# An association between body image dissatisfaction and digit ratio among Chinese children and adolescents

**DOI:** 10.1038/s41598-021-84711-x

**Published:** 2021-03-04

**Authors:** Yongting Yuan, Jingyao Hu, Lili Sun, Yifei Zhang, Bangxuan Wang, Rongying Yao, Hui Han, Lianguo Fu

**Affiliations:** Department of Child and Adolescent Health, School of Public Health, Bengbu Medical College, No. 2600 East Sea Avenue, Bengbu, 233030 Anhui People’s Republic of China

**Keywords:** Psychology, Sexual selection

## Abstract

Body image dissatisfaction (BID) is a negative evaluation of personal physical characteristics, including dissatisfaction with body shape, gender, sexual organs, appearance and so forth, and it plays an important role in growth and development. The second-to-fourth digit ratio (2D:4D) is recognized as a putative indicator of intra-uterine testosterone to estrogen ratio exposure, and it has been observed that higher levels of fetal testosterone exposure are associated with a lower 2D:4D. The present paper contributes to a better understanding of the biological underpinnings of BID by analyzing BID and the digit ratio (2D:4D). We found that the 2D:4D was positively related to appearance dissatisfaction in boys with first spermatorrhea, which means that low prenatal androgen exposure may increase boys’ dissatisfaction with their appearance. In girls with breast development being lower than Tanner stage II, their 2D:4D was negatively related to their body shape dissatisfaction, which means that high prenatal androgen exposure may increase girls’ dissatisfaction with their body shape. These results suggest that the prenatal androgen exposure level might play an important role in the body image dissatisfaction of the offspring.

## Introduction

Body image refers to the sum of an individual's multi-dimensional psychological feelings, such as cognition, attitude, emotion and behaviors about their body shape, appearance, function and so on^1,2^, and it plays an important role in growth and development^3^. The most common body image problem among children and adolescents is body image dissatisfaction (BID), which is a negative evaluation of personal physical characteristics^4^, including dissatisfaction with body shape, gender, sexual organs, and appearance. The study by Li et al.^5^ showed that 59.9% of children and adolescents in China were dissatisfied with their body shape, and the rates of mild and moderate body dissatisfaction were 36.4% and 23.5%, respectively. Fu et al.^6^ found that the body dissatisfaction rate among Chinese children and adolescents was 77.1%. Furthermore, Buckingham-Howes et al.^7^ reported that 60.4% of African American adolescent girls expressed body dissatisfaction and desired to be smaller. A study from Mulasi-Pokhriyal et al.^8^ indicated that 79% of girls and 69% of boys in the United States were dissatisfied with their body. With the rapid increase in children's overweight and obesity worldwide, BID has become an important public health problem affecting the physical and mental health of children and adolescents^9^. The studies have shown that BID is significantly associated with psychological behavioral problems, such as low self-esteem, depression, eating disorders, and suicidal ideation^10–15^, and might increase the risk of hypertension, diabetes, and other chronic diseases^16^.

Puberty, as an important stage of children's growth and development^17^, is accompanied by rapid increases in height and weight, the formation of secondary sexual characteristics and the maturation of sexual organs, the first spermatorrhea or menarche, and increases in gonadal hormone levels^18,19^. It is not only a process of continuous development but also a process of a stage change. However, the physical development of children and adolescents is imbalanced with social adaptation and psychological development, which might cause them to be more prone to develop body image problems.

Recent studies have reported that lower levels of androgen exposure during the fetal period might increase BID during puberty^20,21^. However, the level of intrauterine androgen exposure is difficult to measure directly. The second-to-fourth digit ratio (2D:4D) is measured by dividing the length of the index finger by the length of the ring finger^22^. Manning reported that 2D:4D was a biomarker of prenatal steroid exposure, where a low 2D:4D was associated with prenatal high testosterone and low estrogen exposure^23,24^, and showed that the 2D:4D ratio was negatively correlated with circulating androgens in men and was positively correlated with circulating estrogen in men and women^25^. Some scholars speculate that maternal androgen level during pregnancy may promote the growth of offspring 4D, while maternal estrogen level during pregnancy may promote the growth of offspring 2D^26^. The average finger ratio of men is lower than that of women, that is, a ratio < 1 means that the ring finger is longer, which is called the male pattern^22^. Extensive studies of humans have found correlations between 2D:4D and a variety of physical and psychological conditions, including fertility, risk of cardiovascular disease, social behavior, sexual orientation and exercise ability^27^. However, a meta-analysis by Richards et al. showed that the relationship between sex hormones during pregnancy and 2D:4D is much smaller than that estimated in earlier studies^28^. Other studies suggested that androgen levels during pregnancy are related to gonadal hormone levels in the blood circulation in children^29–31^. The results from Culbert et al.^32^ indicated that sex hormone levels in the blood circulation were related to BID and presented a phased characteristic.

The purpose of this study was to analyze the association between 2D:4D and BID in different pubertal developmental stages and to indirectly show the possible correlation between prenatal hormone exposure and BID in Chinese children and adolescents.

## Materials and methods

### Participants

In this study, a stratified cluster sampling method was used to recruit students aged 8–15 years from two nine-year schools, which were stratified by school and grade, and we took the class as a cluster. First, informed consent was obtained from the students and their guardians. Second, the students who signed the informed consent and met the inclusion criteria were selected as the research participants. Finally, the participants were examined in the third grade A hospital. The inclusion criteria of this study were permanent residence, agreement to sign the informed consent, and no physical disability or mental illness. The exclusion criteria were students with endocrine disease, central nervous system disease, drug-induced secondary obesity, a family history of mental disorders, and other chronic diseases. In this study, there were 571 students aged 8–15 years, including 305 boys (53.4%) and 266 girls (46.6%). This study was approved by The Medical Ethics Committee of the Bengbu Medical College ([2015] No. 003) and conducted in accordance with the Declaration of Helsinki.

### Measures

The index finger (2D) and the ring finger (4D) of the left hand were measured by medical staff who had received standardized training using a Vernier caliper (accurate to 0.01 cm). The finger length was the length from a midpoint of the flexure-crease proximal to the palm to the tip of the finger. After at least 8 h of fasting, venous blood samples (approximately 3 ml) were collected from each participant by the medical staff who had received standardized training. A DFM-96 10 tube radioimmunogamma counter was applied to measure the circulating testosterone and estradiol. The circulating testosterone and estradiol kits were provided by the DIA source company.

### Puberty development

The external genital development and secondary sex characteristics for each participant were evaluated by medical staff who had received standardized training. The boys had their testicular volume measured with a Prader testicular volume meter and were asked whether they had experienced (yes or no) first spermatorrhea. The girls had their breast development checked based on the Tanner stage criteria and were asked whether they had experienced (yes or no) menarche.

According to the Tanner stage criteria^33,34^, secondary sex characteristics development and first spermatorrhea or menarche, the pubertal developmental stages were divided into three stages: stage I (Girls: breast development < Tanner stage II; Boys: testicular volume < 4 ml); stage II (Girls: breast development ≥ Tanner stage II and non-menarche; Boys: testicular volume ≥ 4 ml and non-first spermatorrhea); and stage III (Girls: after menarche; Boys: after first spermatorrhea).

### Body-image dissatisfaction

The teenage body image annoyance questionnaire (TBIAQ)^35^, which is comprised of four cognitive dimensions, including body shape dissatisfaction, gender dissatisfaction, sexual organ dissatisfaction and appearance dissatisfaction, was used to survey BID. Body shape, gender, sexual organ, and appearance dissatisfaction dimensions were assessed by 8, 4, 4, and 9 items, respectively. Each item has three choices, with a score of 1–3, with “1” for “No”, “2” for “Incomplete Yes”, and “3” for “Yes”. Higher scores indicated they were more dissatisfied with their bodies. In this study, the α coefficient for the TBIAQ was 0.889, with values of 0.765, 0.590, 0.701, and 0.875 for body shape, gender, sexual organs, and appearance dissatisfaction dimensions, respectively.

### Statistical analysis

SPSS 23.0 software was used for statistical analysis. The quantitative data were described by Mean ± standard deviation (SD). The differences in age, logarithmic estradiol (lgE2), logarithmic testosterone (lgTTE), 2D, 4D, digit ratio (2D:4D), and BID scores (including body shape, appearance, gender, sexual organs) between the different genders were compared by two-tailed Student’s *t-*test. The differences of the above variables among the different puberty developmental stages were compared by one-way ANOVA (comparisons of two groups using the LSD *t*-test). The Pearson correlation was used to analyze the correlations between lgE2, lgTTE, 2D, 4D, digit ratio (2D:4D) and BID scores (including body shape, appearance, gender, and sexual organs). A multiple linear regression model was used to analyze the above significant factors related to BID after adjusting for ages. The significance level was *P* < 0.05.

## Results

As shown in Table 1, there were significant differences of estradiol, testosterone and 2D:4D between the genders (*P* < 0.05). The results of the one-way ANOVA showed that there were significant differences of ages, lgE2, lgTTE, 2D and 4D among the different pubertal developmental stages (*P* < 0.001); however, there was no significant difference of 2D:4D among the different pubertal developmental stages (*P* > 0.05).

**Table 1:** The comparisons of 2D, 4D, 2D:4D, estradiol and testosterone in gender, puberty developmental stages.

	N	Ages	lgE2	lgTTE	2D (cm)	4D (cm)	2D:4D
**Gender**							
Boys	305	10.94 ± 1.78	0.91 ± 0.57	1.83 ± 0.71	6.40 ± 0.62	6.58 ± 0.64	0.97 ± 0.03
Girls	266	11.20 ± 1.78	1.35 ± 0.61	1.51 ± 0.43	6.40 ± 0.52	6.63 ± 0.52	0.97 ± 0.04
*t*		− 1.759	− 9.045	6.771	− 0.175	− 1.058	2.187
*P*		0.079	< 0.001	< 0.001	0.861	0.291	0.029
**Pubertal developmental stages**							
Stage I	213	9.57 ± 1.19	0.74 ± 0.54	1.27 ± 0.49	5.96 ± 0.45	6.15 ± 0.45	0.97 ± 0.04
Stage II	201	11.28 ± 1.38**	1.12 ± 0.58**	1.90 ± 0.58**	6.53 ± 0.49**	6.73 ± 0.50**	0.97 ± 0.03
Stage III	157	12.81 ± 0.99**^ΔΔ^	1.62 ± 0.41**^ΔΔ^	1.96 ± 0.50**	6.83 ± 0.40**^ΔΔ^	7.05 ± 0.40**^ΔΔ^	0.97 ± 0.03
*F*		326.293	128.981	105.419	178.928	187.491	0.013
*P*		< 0.001	< 0.001	< 0.001	< 0.001	< 0.001	0.987

**Compared with the earlier stage *P* < 0.001; ^ΔΔ^Compared with the early-mid stage *P* < 0.001. Stage I (Girls: breast development < Tanner stage II; Boys: testicular volume < 4 ml); Stage II (Girls: breast development ≥ Tanner stage II and non-menarche; Boys: testicular volume ≥ 4 ml and non-first spermatorrhea); Stage III (Girls: after occurring menarche; Boys: after occurring first spermatorrhea).

**Figure 1:**
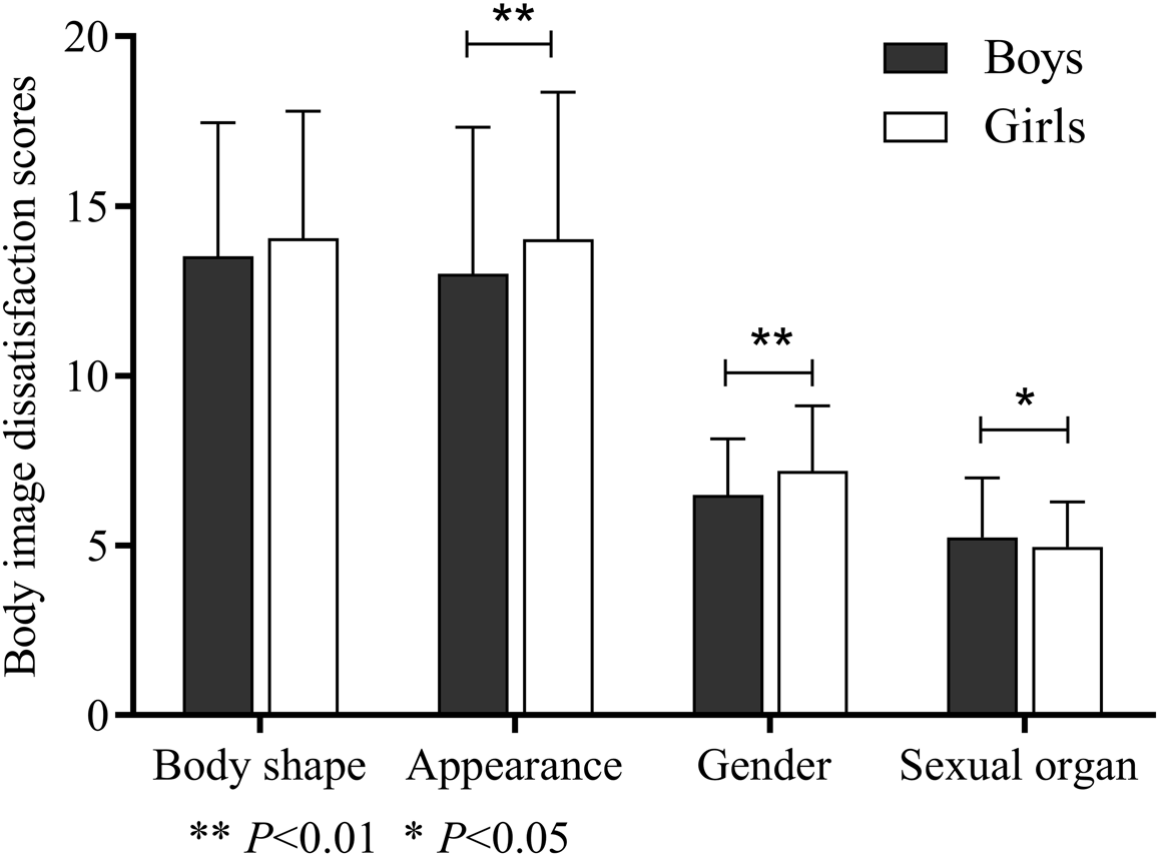
The comparison of body image dissatisfaction scores between genders.

**Figure 2:**
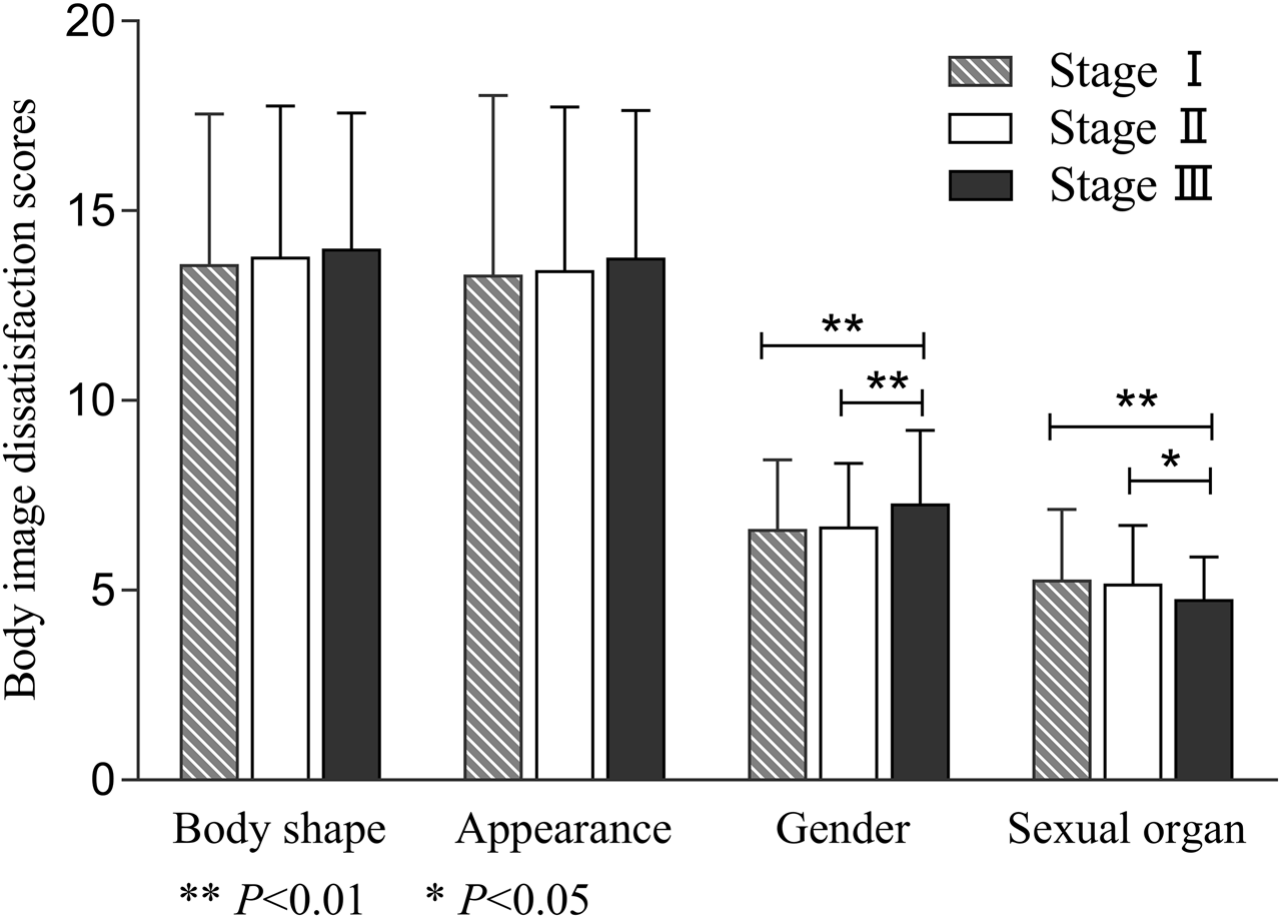
The comparison of body image dissatisfaction scores among puberty developmental stages.

As shown in Fig. 1, the appearance dissatisfaction scores and gender dissatisfaction scores in girls were significantly higher than those in boys (*P* < 0.01), while they were significantly lower in sexual organ dissatisfaction scores (*P* < 0.05). In addition, as shown in Fig. 2, there were significant differences in the gender dissatisfaction scores and sexual organ dissatisfaction scores among different pubertal developmental stages (*P* < 0.01). With puberty developing, the gender dissatisfaction scores were increased and the sexual organ dissatisfaction scores were decreased. There were no significant differences in the body shape dissatisfaction scores and appearance dissatisfaction scores (*P* > 0.05).

**Figure 3:**
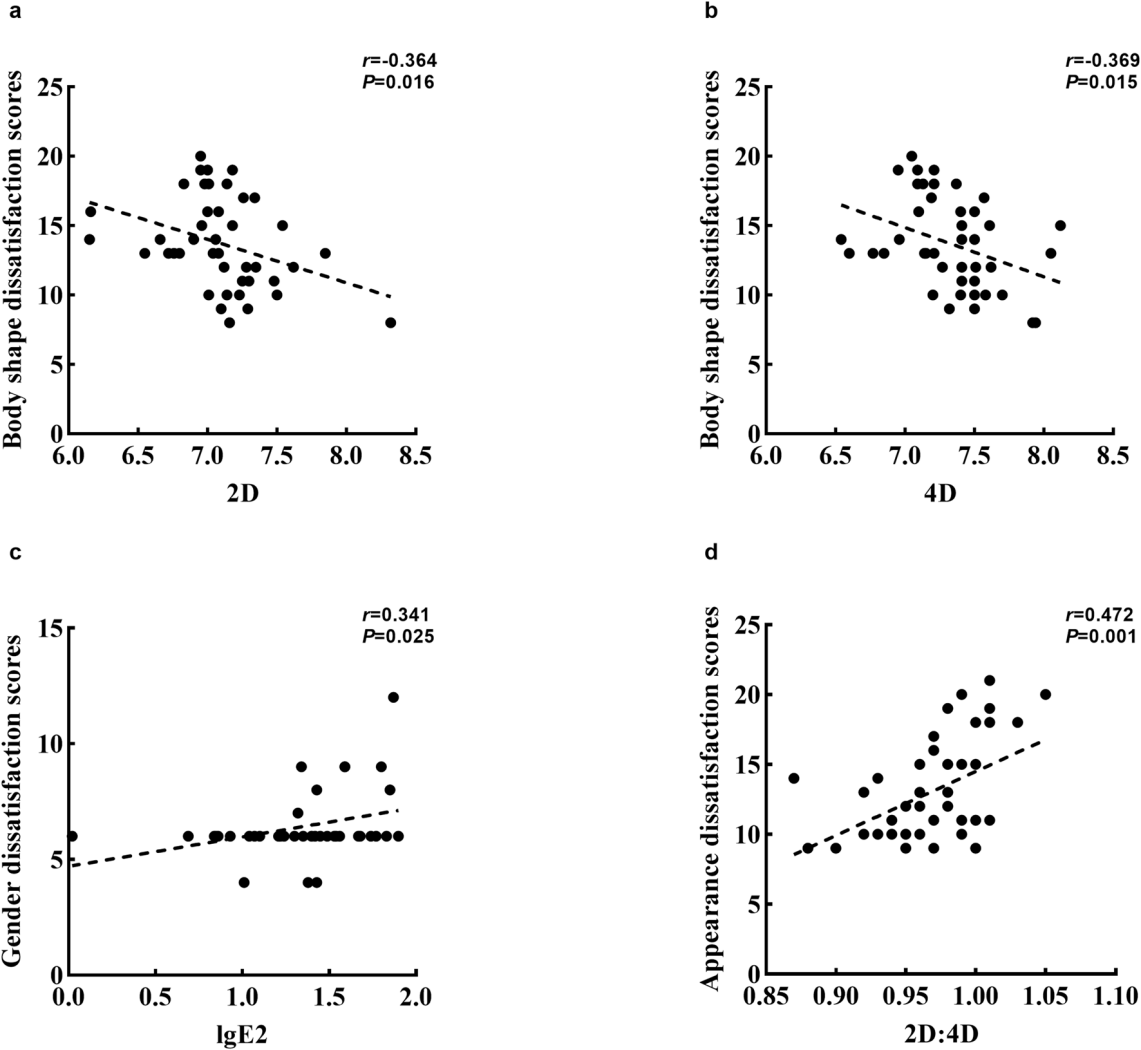
The associations between digit ratio (2D:4D) and body image dissatisfaction among boys with different puberty developmental stages. (**a**) The association between 2D and Body shape dissatisfaction scores among boys with first spermatorrhea; (**b**) the association between 4D and body shape dissatisfaction scores among boys with first spermatorrhea; (**c**) the association between lgE2 and Gender dissatisfaction scores among boys with first spermatorrhea; (**d**) the association between 2D:4D and Appearance dissatisfaction scores among boys with first spermatorrhea.

As shown in Fig. 3, the associations between 2D, 4D and body shape dissatisfaction scores (*r* = − 0.364, *P* < 0.05; *r* = − 0.369, *P* < 0.05 Fig. 3a,b), and between estradiol and gender dissatisfaction scores were significant among boys with first spermatorrhea (*r* = 0.341, *P* < 0.05, Fig. 3c). The 2D:4D was positively related to the appearance dissatisfaction scores among boys with first spermatorrhea (*r* = 0.472, *P* < 0.05, Fig. 3d). Furthermore, there were no significant correlations between lgE2, lgTTE, 2D, 4D, 2D:4D and BID scores (including body shape, appearance, gender, sexual organs) among boys with different pubertal developmental stages as listed in Supplementary Table 1 (*P* > 0.05).

**Figure 4:**
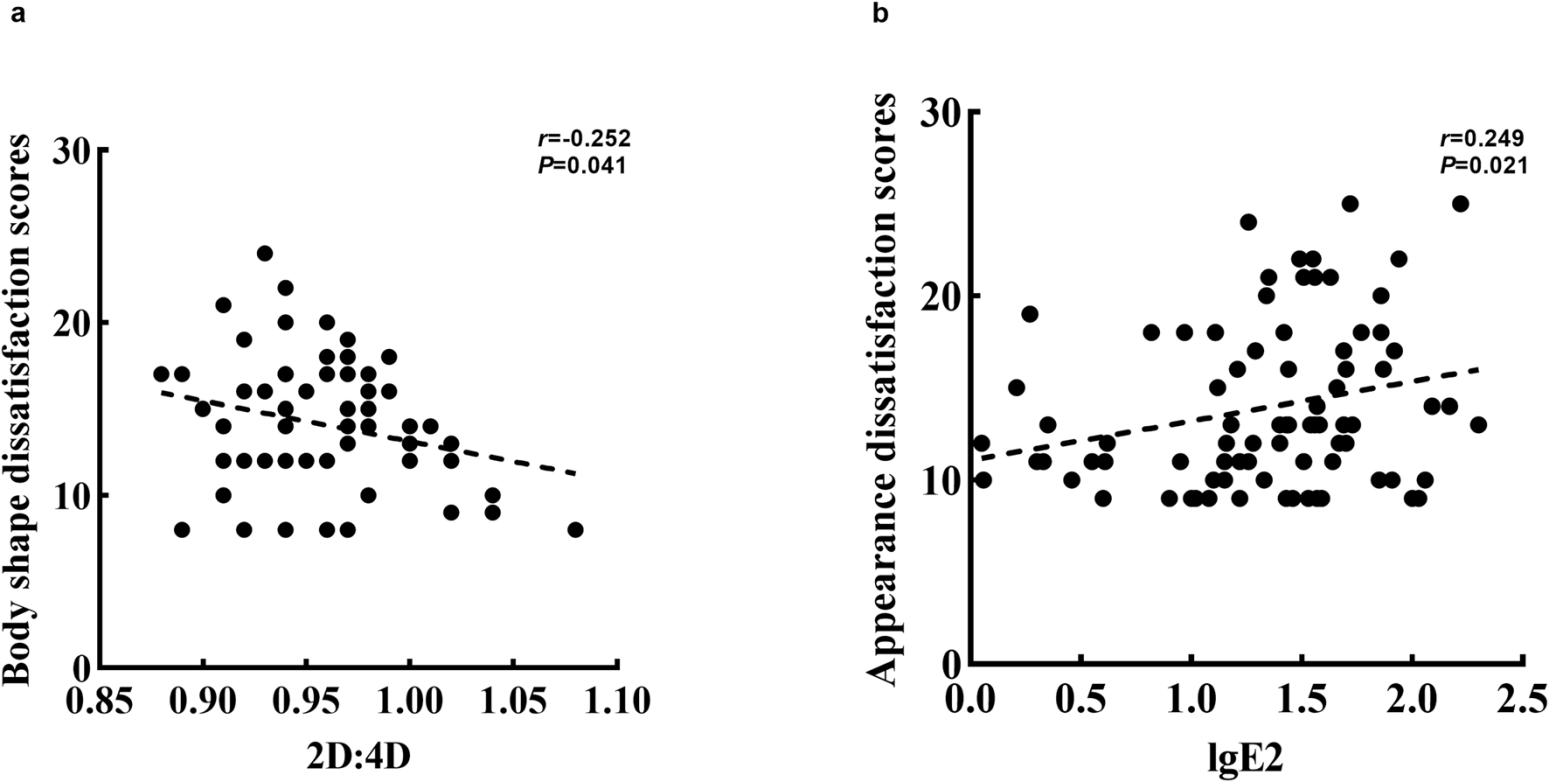
The associations between digit ratio (2D:4D) and body image dissatisfaction among girls with different puberty developmental stages. (**a**) The association between 2D:4D and Body shape dissatisfaction scores among girls with breast development < Tanner stage II; (**b**) the association between lgE2 and Appearance dissatisfaction scores among girls with breast development ≥ Tanner stage II and non-menarche.

As shown in Fig. 4a, the 2D:4D was negatively related to the body shape dissatisfaction scores among girls with breast development being lower than Tanner stage II (*r* = − 0.252, *P* < 0.01). Estradiol was positively correlated with the appearance dissatisfaction scores among girls with breast development ≥ Tanner stage II and non-menarche (*r* = 0.249, *P* < 0.05, Fig. 4b). Furthermore, there were no significant correlations between lgE2, lgTTE, 2D, 4D, 2D:4D and BID scores (including body shape, appearance, gender, sexual organs) among girls with different pubertal developmental stages (listed in Supplementary Table 2) (*P* > 0.05).

As shown in Supplementary Table 3, there were significant positive associations between 2D:4D and appearance dissatisfaction scores (β(SE) = 46.10(13.55), *P* = 0.002), and between estradiol and gender dissatisfaction scores after adjusting for ages in boys with first spermatorrhea (β(SE) = 1.35(0.57), *P* = 0.022); however, the 2D and 4D were not significantly related to the body shape dissatisfaction scores (*P* > 0.05). As shown in Supplementary Table 4, the 2D:4D was significantly related to body shape dissatisfaction scores after adjusting for ages in girls with breast development being lower than Tanner stage II (β(SE) = − 23.99(10.88), *P* = 0.031), and estradiol was significantly correlated with appearance dissatisfaction scores after adjusting for ages in girls with breast development ≥ Tanner stage II and non-menarche (β(SE) = 1.98(0.94), *P* = 0.038).

## Discussion

The present study showed that the gender and appearance dissatisfaction scores in girls were significantly higher than those in boys, which indicates girls were more dissatisfied with their gender and appearance. One of reasons might be that some parents or family members prefer boys^36–38^. In addition, the studies generally show that, in comparison to boys, girls pay more attention to their appearance, compare their appearance more with that of their peers, and spend more time on social network sites^39,40^.

The results of the current study showed that the sexual organ dissatisfaction scores in girls were significantly lower than those of boys, which indicated that boys were more dissatisfied with their sexual organs. Boys might pay more attention to the development of their sexual organs than girls do, and talk more frequently about them with their friends, which might be one of the reasons why boys are dissatisfied with their genitals.

It is well known that the breast development (Tanner stage II) or testicular volume (4 ml) is a sign of starting puberty, and the occurrence of menarche or first spermatorrhea means that the sexual organs are mature among girls or boys, respectively. The present study showed that gender dissatisfaction increased gradually during pubertal development, which indicated that boys and girls were more dissatisfied with their gender after the occurrence of menarche or first spermatorrhea. The gender recognition in boys and girls during the prepuberty stage might be only from a biological point of view, and they might not recognize the social attributes of gender^41–43^. With the development of the sexual organs and the recognition of the social attributes of gender, they may begin to accept the development and maturity of their sexual organs, and also to realize the external evaluation of themselves^44^.

The present study showed that estradiol was positively correlated with gender dissatisfaction scores in boys with first spermatorrhea, which indicated that higher levels of estradiol might lead to higher gender dissatisfaction. The increased estradiol might be related to increased gender dissatisfaction by leading to a more feminine body type (which a male-typical teenager may be uncomfortable with) or with a gender identity less typical of a male teenager (or both)^45^.

The results of the present study also showed that 2D:4D was positively correlated with appearance dissatisfaction scores in boys with first spermatorrhea, which indicated that boys with a higher 2D:4D might be more dissatisfied with their appearance. The previous studies showed that a higher 2D:4D indicated a lower exposure level to androgens during pregnancy^24^. The studies based on finger length suggested that androgen exposure during pregnancy might be positively correlated with androgen levels in the offspring, with lower androgen exposure during pregnancy leading to lower androgen and higher estrogen levels in the offspring^46,47^, which might lead to dissatisfaction with their appearance among boys in the late puberty stage. The evidence^20,21^ shows that lower levels of androgen exposure during the fetal period might increase puberty BID. The results of this study showed that there were no associations between body shape dissatisfaction and 2D:4D in boys. Usually, the most direct factor affecting children's body shape dissatisfaction is obesity, but the study shows that 2D:4D is not associated with obesity^48^.

In contrast to the above findings, the present study also showed that 2D:4D was negatively related to the body shape dissatisfaction scores among girls with breast development < Tanner stage II, which indicated that the girls with a lower 2D:4D might be more dissatisfied with their body shape. Girls with higher testosterone might develop more facial and body hair in their prepubertal stage than others, and develop larger muscles, causing girls to be dissatisfied with their bodies. In addition, testosterone is a precursor of estradiol, which may lead to fat deposits in the hips and buttocks^49^. Research by Noha et al.^22^ showed that females with BMI ≥ 25 have more C patterned hands (the index finger is longer).

There were no significant correlations between sexual organ dissatisfaction, gender dissatisfaction and digit ratio among the girls in the current study. This might be related to the young age of the children in the study sample. In addition, in most countries, sex education is compulsory, with more than half of school biology courses dealing with adolescent-related topics such as physiology and reproduction^50^. This might be one of the reasons why the girls in this study have a correct understanding of their gender and sexual organs.

There were several limitations. First, this study only measured the children’s left hand digital length, and their right hand digital length should be measured in future studies to verify the results. Second, the pubertal development stages were defined based on cross-sectional data, which meant that the comparison of body image dissatisfaction among the different pubertal stages was influenced by their birth dates. Third, based on this cross-sectional study, we observed the association between 2D:4D and body image dissatisfaction, however, the vast majority of effects observed in this study were not statistically significant, and that those that were might therefore simply be chance findings (i.e., Type 1 errors) and so need to be replicated for firm conclusions to be drawn. In addition to biological factors promoting a correlation between body image dissatisfaction and the outcome indicators described in this article, environmental and social factors may also play a role in these associations.

## Conclusion

In summary, the present study showed the 2D:4D was positively related to appearance dissatisfaction among boys with first spermatorrhea and negatively associated with body shape dissatisfaction among girls with breast development < Tanner stage II, suggesting that prenatal androgen exposure might play an important role in body image dissatisfaction. However, these findings need to be further verified.

## Supplementary Information

Supplementary Table 1.

Supplementary Table 2.

Supplementary Table 3.

Supplementary Table 4.
