# Supplementary material for: An association between body image dissatisfaction and digit ratio among Chinese children and adolescents
Source: Sci Rep. 2021 Mar 4;11:5217. doi: 10.1038/s41598-021-84711-x (PMC7970844; doi:10.1038/s41598-021-84711-x)
Supplement: Supplementary file 1 — Supplementary Table 1. [file 41598_2021_84711_MOESM1_ESM.pdf]

Supplementary table 1 The associations between digit ratio (2D:4D) and body image dissatisfaction among boys with different puberty developmental stages

Variables	Body shape		Gender		Sexual organ		Appearance	
	r	*P*	*r*	*P*	*r*	*P*	*r*	*P*
Stage I								
lgE2	0.132	0.112	-0.021	0.801	0.001	0.987	0.101	0.224
lgTTE	0.040	0.634	-0.072	0.386	-0.024	0.776	0.093	0.265
2D(cm)	0.095	0.252	-0.111	0.179	-0.041	0.620	0.005	0.952
4D(cm)	0.061	0.465	-0.156	0.058	-0.031	0.708	-0.027	0.747
2D:4D	0.090	0.280	0.087	0.294	-0.016	0.847	0.067	0.423
Stage II								
lgE2	-0.009	0.921	0.046	0.622	0.037	0.691	-0.033	0.727
lgTTE	0.134	0.152	0.120	0.200	-0.038	0.689	0.052	0.578
2D(cm)	0.108	0.253	0.180	0.054	0.028	0.769	0.032	0.732
4D(cm)	0.099	0.291	0.154	0.099	-0.039	0.682	0.053	0.571
2D:4D	0.042	0.654	0.077	0.415	0.149	0.113	-0.025	0.788
Stage III								
lgE2	0.277	0.072	0.341	0.025	0.164	0.295	0.191	0.220
lgTTE	-0.083	0.599	-0.144	0.358	-0.106	0.497	-0.084	0.593
2D(cm)	-0.364	0.016	-0.049	0.753	0.058	0.709	0.167	0.284
4D(cm)	-0.369	0.015	-0.059	0.707	-0.088	0.573	-0.202	0.194
2D:4D	-0.054	0.730	0.007	0.964	0.194	0.213	0.472	0.001

Note. Stage I: testicular volume < 4 ml; Stage II: testicular volume ≥ 4 ml and non-first spermatorrhea; Stage III: after occurring first spermatorrhea.
