# Supplementary material for: An association between body image dissatisfaction and digit ratio among Chinese children and adolescents
Source: Sci Rep. 2021 Mar 4;11:5217. doi: 10.1038/s41598-021-84711-x (PMC7970844; doi:10.1038/s41598-021-84711-x)
Supplement: Supplementary file 2 — Supplementary Table 2. [file 41598_2021_84711_MOESM2_ESM.pdf]

Supplementary table 2 The associations between digit ratio (2D:4D) and body image dissatisfaction among girls with different puberty developmental stages

Variables	Body shape		Gender		Sexual organ		Appearance	
	r	*P*	*r*	*P*	*r*	*P*	*r*	*P*
Stage I								
lgE2	0.092	0.463	-0.087	0.485	-0.060	0.634	-0.022	0.859
lgTTE	0.047	0.710	0.151	0.227	0.059	0.638	0.166	0.182
2D(cm)	-0.012	0.921	0.071	0.570	-0.140	0.261	-0.061	0.629
4D(cm)	0.127	0.311	0.046	0.712	-0.083	0.506	0.041	0.746
2D:4D	-0.252	0.041	0.034	0.785	-0.106	0.396	-0.181	0.146
Stage II								
lgE2	0.105	0.338	0.058	0.596	0.106	0.330	0.249	0.021
lgTTE	0.031	0.778	0.128	0.240	0.083	0.446	-0.020	0.855
2D(cm)	-0.055	0.617	0.053	0.629	0.051	0.642	0.046	0.676
4D(cm)	-0.049	0.653	0.082	0.450	-0.021	0.847	0.061	0.578
2D:4D	-0.028	0.797	-0.067	0.541	0.120	0.270	-0.039	0.720
Stage III								
lgE2	0.048	0.614	0.108	0.251	-0.010	0.918	0.072	0.448
lgTTE	0.053	0.573	0.077	0.415	-0.002	0.983	0.069	0.467
2D(cm)	-0.008	0.936	-0.174	0.063	-0.150	0.112	0.128	0.176
4D(cm)	0.007	0.937	-0.136	0.149	-0.088	0.349	0.100	0.292
2D:4D	-0.022	0.816	-0.061	0.517	-0.092	0.330	0.044	0.644

Note. Stage I: breast development < Tanner stage II; Stage II: breast development ≥ Tanner stage II and non-menarche; Stage III: after occurring menarche.
