# Supplementary material for: An association between body image dissatisfaction and digit ratio among Chinese children and adolescents
Source: Sci Rep. 2021 Mar 4;11:5217. doi: 10.1038/s41598-021-84711-x (PMC7970844; doi:10.1038/s41598-021-84711-x)
Supplement: Supplementary file 3 — Supplementary Table 3. [file 41598_2021_84711_MOESM3_ESM.pdf]

Supplementary table 3 The results of associations between 2D, 4D, estradiol, 2D:4D and BID scores using the multiple linear regression in boys with stage III

Independent variables	*β*	*SE*	*t*	*P*	*β(95%CI)*	
					Lower	Upper
Model 1 (association between 2D,4D and body shape dissatisfaction scores in boys with stage III)						
age	0.604	0.564	1.072	0.290	-0.536	1.745
2D	-1.571	1.784	-0.880	0.384	-5.179	2.038
4D	-2.467	2.003	-1.232	0.225	-6.518	1.584
Model 2 (association between estradiol and gender dissatisfaction scores in boys with stage III)						
age	-0.164	0.253	-0.648	0.521	-0.675	0.347
IgE2	1.353	0.565	2.393	0.022	0.210	2.496
Model 3 (association between 2D:4D and appearance dissatisfaction scores in boys with stage III)						
age	0.176	0.583	0.302	0.764	-1.002	1.353
2D:4D	46.095	13.545	3.403	0.002	18.720	73.469

Note. Stage III: after occurring first spermatorrhea.
