# Supplementary material for: An association between body image dissatisfaction and digit ratio among Chinese children and adolescents
Source: Sci Rep. 2021 Mar 4;11:5217. doi: 10.1038/s41598-021-84711-x (PMC7970844; doi:10.1038/s41598-021-84711-x)
Supplement: Supplementary file 4 — Supplementary Table 4. [file 41598_2021_84711_MOESM4_ESM.pdf]

Supplementary table 4 The results of associations between 2D:4D, estradiol and BID scores using the multiple linear regression in girls

	β	*SE*	*t*	*P*	*β(95%CI)*	
					Lower	Upper
Model 1(association between 2D:4D and body shape dissatisfaction scores in girls with stage I)						
age	1.025	0.394	2.601	0.012	0.237	1.812
2D：4D	-23.994	10.882	-2.205	0.031	-45.739	-2.249
Model 2 (association between estradiol and appearance dissatisfaction scores in girls with stage II)						
age	0.250	0.402	0.622	0.535	-0.549	1.050
lgE2	1.979	0.937	2.113	0.038	0.116	3.841

Note. Stage I: breast development < Tanner stage II; Stage II: breast development ≥ Tanner stage II and non-menarche.
